# Atypical presentation of γ/δ mycosis fungoides with an unusual phenotype and *SOCS1* mutation

**DOI:** 10.1515/biol-2022-0925

**Published:** 2024-07-30

**Authors:** Pia Rude Nielsen, Lone Schejbel, Pär Lars Josefsson, Lone Skov, Signe Ledou Nielsen

**Affiliations:** Department of Pathology, Herlev and Gentofte Hospital, Copenhagen University Hospital, Borgmester Ib Juuls Vej 73, Staircase 7, floor 4 (L5), 2730 Herlev, Copenhagen, Denmark; Department of Hematology, Rigshospitalet, Copenhagen University Hospital, Copenhagen, Denmark; Department of Dermatology and Allergy, Herlev and Gentofte Hospital, Copenhagen University Hospital, Copenhagen, Denmark; Department of Clinical Medicine, University of Copenhagen, Copenhagen, Denmark; Department of Pathology, Herlev and Gentofte Hospital, Copenhagen University Hospital, Copenhagen, Denmark

**Keywords:** cutaneous T-cell lymphoma, mycosis fungoides, immunophenotypic, gamma/delta phenotype

## Abstract

Mycosis fungoides is the most frequent subtype of primary cutaneous T-cell lymphomas. The diagnosis is based on a thorough clinic-pathologic correlation, which can, especially in early-stage disease, be challenging due to similarities with several benign skin disorders such as psoriasis and atopic dermatitis. Here, we present a case of an 81-year-old man with a 20-year-long medical history of skin problems treated as psoriasis with limited effect. Since December 2021, the patient experienced worsening of his skin symptoms with rapidly growing tumors and widespread patches and plaques. Positron emission tomography/computed tomography evaluation revealed markedly metabolic activity related to the skin tumors and increased FDG uptake in several retroperitoneal lymph nodes. Histological assessment of skin biopsies demonstrated a highly proliferative T-cell lymphoma with a γ/δ+ and CD8+ cytotoxic phenotype. The morphology of the tumor cells appeared blastic with an abnormal immunephenotype CD3+, CD2−, CD5_dim_, CD4−, CD8+, CD56−, and CD30−. Next-generation sequencing detected a likely pathogenic *SOCS1* mutation with an allele frequency of 72% as well as a *STAT3* variant of unknown significance. This case highlights the diagnostic complexity of an indolent skin lymphoma evolving into an aggressive cytotoxic lymphoma.

## 1 Background

Mycosis fungoides (MF) is the most common subtype of cutaneous T-cell lymphomas (CTCLs) and is usually considered as slowly progressing with an indolent disease course [1]. Although MF is considered indolent, a subset of patients experience disease encompassing ulcerating tumors, which in some cases transform into large-cell lymphoma, that may be either CD30 positive or CD30 negative [2]. The most common immunephenotype of MF is CD3+, CD4+/CD8−, and T-cell receptor alphabeta+ (TCRαβ), though in rare cases of otherwise classical MF a CD4−/CD8+ as well as TCRγ/δ+ phenotype can be seen [3,4].

However, the recognition of CTCLs with an γ/δ+ phenotype has increased with the availability of TCRγ/δ immunohistochemical analyses in paraffin sections, leading to a prognostically more heterogeneous group of primary cutaneous γ/δ T-cell lymphomas (PCGDTLs), including γ/δ+ MF [4,5,6,7].

Here, we describe an atypical presentation of MF, clinically interpreted and treated as psoriasis through two decades, which suddenly transformed into a highly aggressive γ/δ T-cell lymphoma, leading to death 3 months later.

## 2 Case presentation

**Figure 1:**
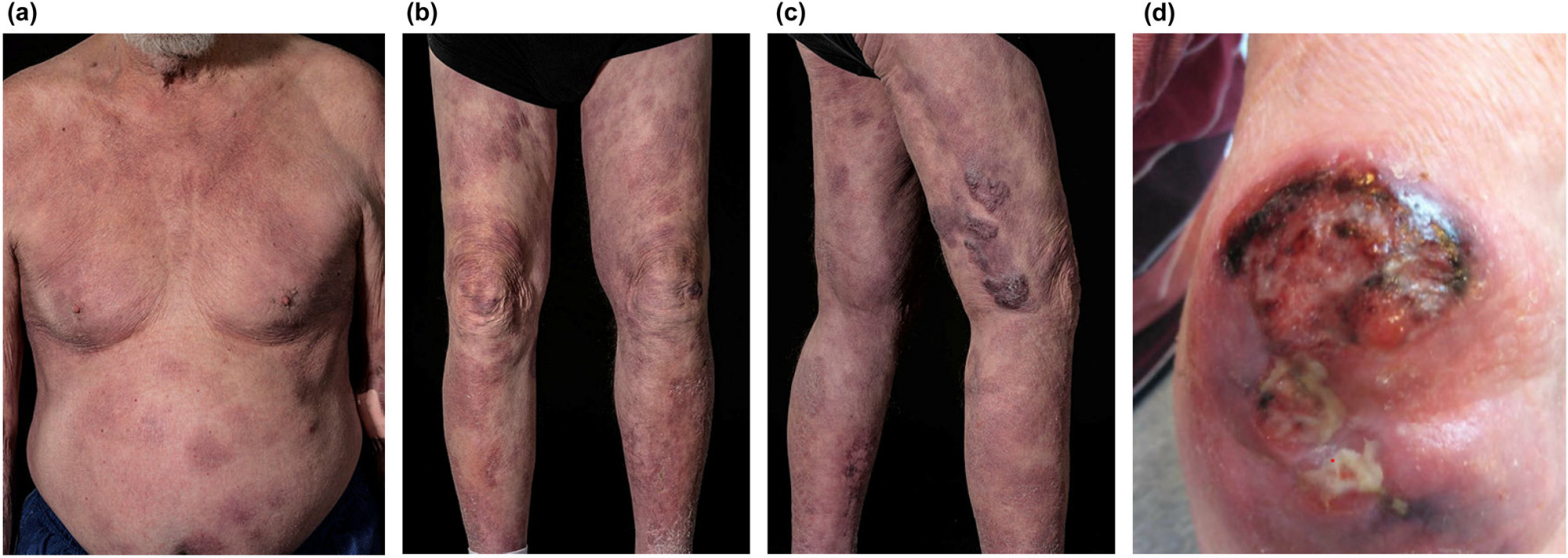
Clinical presentation. (a–c) Widespread patches, plaques, and tumors involving the trunk and lower extremities. (d) Ulcerated tumor lesion on the left elbow.

An 81-year-old man with a 20-year-long medical history of scaly plaques on his legs and back, which was clinically interpreted as psoriasis without histopathological confirmation. The skin lesions had through the years been treated with topical steroids and ultraviolet B irradiation, although with limited effect. In December 2021, the patient experienced worsening of his skin symptoms and developed rapidly growing tumors on the upper extremities. A punch biopsy was taken from the tumor lesion at the left elbow, which demonstrated a highly proliferative T-cell lymphoma with γ/δ+ and CD8+ cytotoxic phenotypes. The patient was referred to the hospital and clinical examination showed widespread, partly confluent patches and indurated plaques with ulcerating tumors on both elbows (Figure 1). No enlarged superficial lymph nodes were detected upon physical examination. The patient had no B symptoms such as fever, night sweats, or unintentional weight loss, but he had noticed poorer wound healing over the past year.

**Figure 2:**
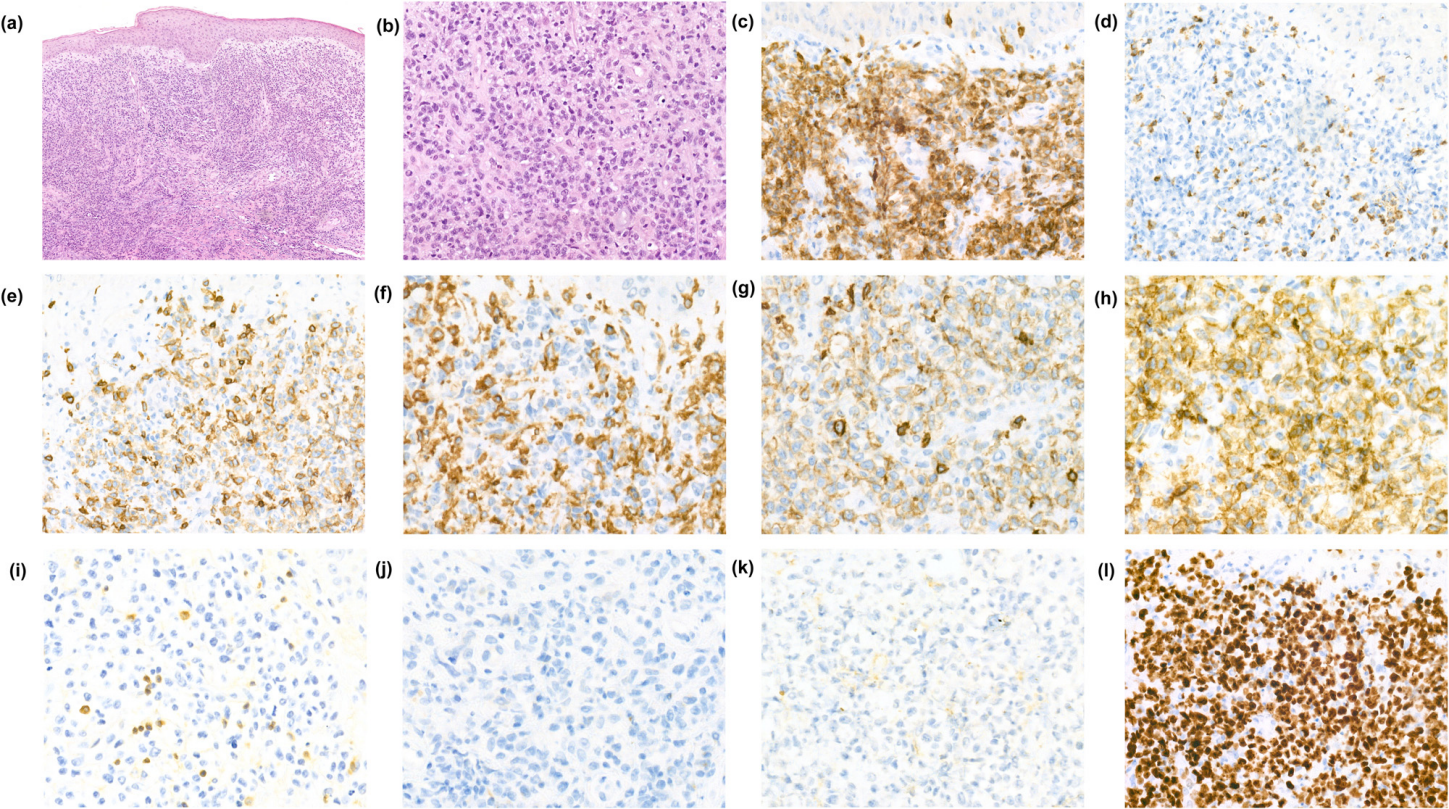
Histopathological examination of the tumor lesion on the left elbow. (a) and (b) H&E-stained slide with a deep dermal infiltrate of atypical medium to large-sized lymphocytes. Immunhistochemical analysis demonstrated the tumor infiltrate consisted of (c) CD3 (+), (d) CD2 (−), (e) CD5_dim_, (f) CD4 (−), (g) CD8 (+), (h) TCRγ (+), (i) TCRαβ (−), (j) CD30 (−), and (k) CD56 (−) T lymphocytes with a high proliferation index (l) Ki67 (∼100%). Magnification (a) 10×, (b–e, l) 40×, and (f–k) 63×.

The initial skin biopsy from the ulcerating tumor at the left elbow as well as a skin biopsy taken from the tumor at the right upper arm showed a dense dermal infiltrate composed of medium to large-sized atypical, blastic-appearing lymphocytes with prominent nucleoli and sparse cytoplasm. There were frequent mitoses and no necrosis, and the tumor infiltrate showed an angio-destructive pattern with scattered small areas of hemorrhage. The epithelium was well differentiated, and no sign of epidermotropism was noted. Immunohistochemically the tumor-forming infiltrate consisted of T cells expressing CD3, CD8, TIA-1, and strong expression of TCRγ (clone H-41) [8]. The γ/δ+ lymphocytic tumor cells displayed a complete loss of CD2, partial downregulation of CD5, and showed no expression of CD4, CD30, CD56, TdT, or TCRαβ (clone 8A3/BetaF1) (Figure 2). c-Myc was positive in approximately 70% of the atypical lymphocytes and the proliferation marker Ki67 was near 100%. Epstein-Barr virus-encoded small RNA *in situ* hybridization was negative.

Additional biopsies from infiltrative patches and plaques at the left shoulder and lower back demonstrated a modest epidermotropism and scattered atypical T lymphocytes with the same abnormal phenotype in a reactive CD4+ and TCRαβ+ dermal infiltrate.

**Figure 3:**
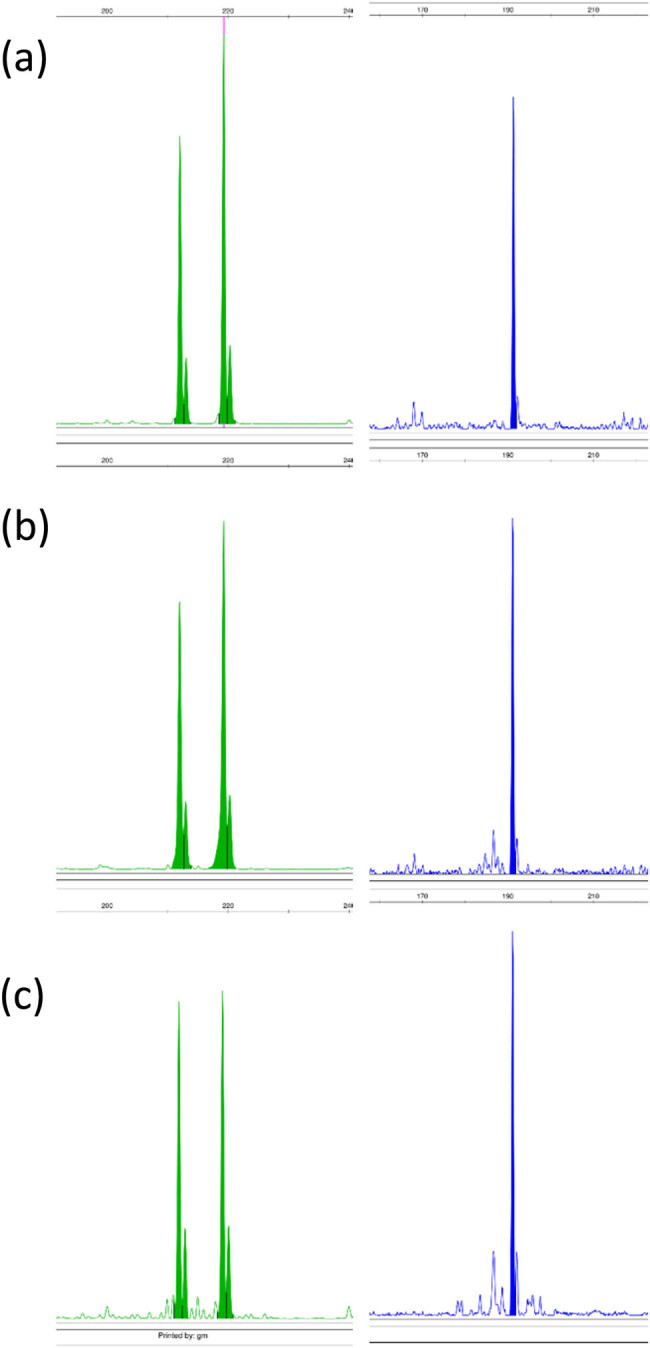
Gene rearrangement analysis of *TCRG Vγ1-8/Jγ* (green) and *TCRB Dβ/Jβ* (blue) showed a monoclonal T-cell population with identical basepair peaks in the initial tumor biopsy and subsequent biopsies. (a) Onset tumor biopsy from the left elbow. (b) Tumor lesion right elbow. (c) Patch lesion on the left shoulder.

Clonal rearrangements were detected using IdentiClone TCRB + TCRG T-Cell Clonality Assay, ABI Fluorescence Detection (Invivoscribe). Identical clonal *TCRG* (Vγ1-8/Jγ) and an incomplete *TCRB* (Dβ/Jβ) rearrangement were found in the initial and subsequent biopsies (Figure 3).

Next-generation sequencing (NGS) was performed with a custom-designed panel as previously described [9]. A likely pathogenic *SOCS1* mutation (NM_003745.1, c.16C>T, p.(Gln6Ter)) with an allele frequency of 72% (loss of function), as well as a *STAT3* variant (NM_139276.2, c.831_832delCCinsTT, p.(Arg278Cys)) of unknown significance, was detected.

Two histological differential diagnoses were considered; an indolent course of primary cutaneous γ/δ+ T-cell lymphoma, although they usually are CD4−/CD8− and express CD56, or a rare presentation of transformed, CD30− MF with a CD8+ and TCRγ/δ+ phenotype. The medical history and clinical presentation supported the diagnosis of atypical MF with aggressive transformation.

After histological diagnosis, the patient underwent staging investigations with whole-body positron emission tomography and computed tomography (PET–CT), peripheral blood chemistry as well as bone marrow aspirate and biopsy. PET–CT revealed markedly metabolic activity related to the skin tumors and increased FDG uptake in several retroperitoneal, iliac, and inguinal lymph nodes. Histopathological examination of an inguinal lymph node showed reactive changes compatible with dermatopathic lymphadenopathy and no clonal TCR rearrangements were detected. Bone marrow examination was normal and blood chemistry revealed mild macrocytic anemia and slightly elevated leucocytes with neutrophilic granulocytosis. Serum lactate dehydrogenase was within the normal reference range. Viral serology was normal/negative.

The patient started treatment with whole body irradiation (planned for 1 Gy/4 times a week, in total 20 treatments) with an immediate effect on the tumors on the elbows. Unfortunately, the patient experienced rapidly growing tumors in other areas of the skin, and the treatment was therefore stopped. The patient was shortly after the last radiation dose hospitalized with severe sepsis and died within a few days. No autopsy was performed.


**Informed consent:** Informed consent has been obtained from all individuals included in this study.
**Ethical approval:** The research related to human use has been complied with all the relevant national regulations, institutional policies, and in accordance with the tenets of the Helsinki Declaration, and has been approved by the authors’ institutional review board or equivalent committee.

## 3 Discussion

In this case report, we describe an atypical presentation of a γ/δ+ MF-case, initially clinically perceived as psoriasis with a two-decade-long indolent disease course.

The skin lesions developed into an aggressive lymphoma characterized by the progression of rapid growing, ulcerating tumors with a CD8+ cytotoxic and γ/δ phenotype with CD30− transformation and *SOCS1* mutation.

MF with a γ/δ phenotype is rare and mainly reported with an initial indolent disease course that eventually, for the majority of cases, develops into an aggressive cytotoxic lymphoma [10,11]. Currently, it is not possible to predict which γδMF case will develop into an aggressive PCGDTCL-like clinical phenotype.

PCGDTLs are a heterogeneous group of rare lymphomas and are primarily known for their aggressive disease course with a poor prognosis [7]; however, several indolent PCGDTCL cases have been reported, in particular MF-like cases [6,10,12]. Interestingly, different subsets of PCGDTLs related to distinct cells of origin for epidermotropic (Vδ1) vs dermal/subcutaneous (Vδ2) PCGDTL have been demonstrated [13] and it appears that the predominantly epidermotropic variant portends a better prognosis than the dermal/subcutaneous presentation [5,11]. Accordingly, an adequate interpretation of γδ+ CTCLs needs a close correlation with histopathological and clinical findings, as a γδ phenotype does not necessarily imply a more aggressive clinical behavior as it is also observed in benign T-cell lymphoid proliferations of the skin [7].

The diagnostic challenge of determining whether the epidermotropic variant of primary cutaneous γ/δ T-cell lymphomas (PCGDTLs) presenting with patches and plaques should be classified as indolent PCGDTCL or mycosis fungoides (MF) with a γ/δ+ phenotype remains to be clarified. The current WHO-EORTC classification recognizes different subsets of PCGDTL but suggests that cases with an MF-like clinical- and histological presentation should be classified as MF irrespective of phenotype [1].

In our case, two histological differential diagnoses were considered: an indolent course of PCGDTCL or a rare presentation of CD30− transformed γ/δ MF. The clinical manifestation of patches and plaques, concomitantly with the prolonged indolent disease course, aligns with the typical presentation of MF. However, the histopathological findings posed a challenge due to the rarity of MF displaying a CD8+ cytotoxic and γδ+ phenotype, along with CD30− large cell transformation. Traditionally, the immunohistochemical phenotype of MF exhibits CD3+, CD4+/CD8−, often displaying varying loss of CD2, CD5, and CD7 during disease progression [3]. Although a majority of MF cases with large cell transformation usually express CD30 instances of transformed CD30− MF have been documented. Notably, CD30− transformed MF cases indicate a twofold higher hazard ratio of mortality compared to CD30+ MF [14].

PCGDTCLs predominantly exhibit CD4−/CD8− phenotype, commonly displaying loss of CD5, despite occasional reports of CD8+ PCGDTCL cases [7]. The neoplastic T cells typically express CD56 and are mostly positive for cytotoxic markers such as TIA-1 and granzyme B.

In our case, the morphology of the neoplastic lymphocytes displayed a blastic appearance, manifesting as a deep, dense dermal infiltrate with an angio-destructive pattern and hemorrhage, often observed in PCGDTL. However, distinct features such as necrotic keratinocytes and interface changes were absent. The NGS analysis results, unfortunately, did not significantly contribute to our differential diagnostic considerations. Although detection of mutation in the tumor suppressor *SOCS1* gene stands as one of the most prevalent genetic alterations in MF, this genetic abnormality is also shared by other primary cutaneous lymphomas [15,16]. The expression of CCR4 might have potentially aided in our differential diagnostic considerations given that MF typically demonstrates strong positivity for CCR4, whereas CCR4 is largely absent in PCGDTLs [7,17,18]. Unfortunately, this analysis was not feasible in our case.

Regrettably, the absence of prior biopsies hindered our ability to retrospectively analyze the disease evolution, given the patient´s initial clinical diagnosis of psoriasis without any pathological assessment. Consequently, an essential inquiry arises: did the patient initially manifest an atypical, protracted, and clinically indolent form of γδMF or PCGDTCL, or was there an undisclosed coexistence of MF and psoriasis?

In summary, our case represents a unique and uncommon occurrence of γ/δ+ MF, characterized by a prolonged, indolent clinical course that ultimately culminated in the development of a highly aggressive cytotoxic lymphoma. This case emphasizes the significance of comprehensive clinical interpretation and meticulous pathological assessment during the initial presentation.
